# Predictors of perceptions of human rights violations during the Chilean social outburst of 2019

**DOI:** 10.3389/fpsyg.2023.1133428

**Published:** 2023-04-28

**Authors:** Silvia F. Carrasco Paillamilla, Rodolfo Disi Pavlic

**Affiliations:** ^1^Faculty of Education, University of Concepcion, Concepción, Chile; ^2^Department of Sociology, Political Science and Public Administration, Temuco Catholic University, Temuco, Araucania, Chile; ^3^Department of Political Studies, University of Santiago, Santiago, Chile

**Keywords:** human rights, public opinion, protests, Chile, political perceptions

## Abstract

The 2019 social outburst in Chile marked a significant turning point for the nation’s politics and society, with widespread reports of human rights violations committed by the armed forces and the Police during demonstrations and riots. Despite the attention given to these events, few studies have systematically analyzed perceptions of human rights violations in such contentious contexts. To investigate the factors influencing perceptions of human rights violations during the 2019 Chilean social outburst, we conducted ordered logistic regressions using data from a nationally representative survey fielded during the unrest. Our findings reveal that participation in demonstrations, use of social media for political information, fear of crime, and proximity to violent protests are correlated with the perception that security forces frequently violated human rights during the outburst. These results contribute to the understanding of public perceptions of human rights violations in the context of the 2019 Chilean social outburst and provide insights for future research on the role of individual and contextual factors in shaping these perceptions.

## 1. Introduction

The social unrest that began on Friday, October 18, 2019, in Chile made headlines around the world. The protests, initially started by high school students, escalated into widespread looting and arson, leading to the declaration of a state of emergency in Santiago. The unrest quickly spread to a dozen other cities, with at least five subway stations and buses set ablaze, and violent demonstrators looting supermarkets and pharmacies, as tanks were deployed to maintain order in Santiago’s Plaza Baquedano (Bonnefoy, 2019). The level of violence and lawlessness on the streets, unheard of since Chile’s return to democracy in 1990, perplexed both domestic and international observers, many of whom considered Chile to be and “oasis” of stability in Latin America (Somma et al., 2021). Political reactions were varied, ranging from an initial government perception that gangs and foreign agents had orchestrated the unrest (Dammert and Sazo, 2020) to a package of policy concessions (Deutsche Welle, 2019) and, a (ultimately failed) constitution-making process to replace the current, dictatorship-era Constitution (Heiss, 2021).

However, the country also suffered unprecedent levels of human rights violations committed by the armed forces and *Carabineros* (police). Reports by the National Institute of Human Rights (2020) indicate that 3,442 individuals, including 254 children, have been seriously injured as a result of police repression, with 1,500 cases of human rights violations. Of these injuries, 1,974 were caused by bullets or rubber bullets, resulting in 347 eye injuries. These statistics have been corroborated by several international organizations (Sehnbruch and Donoso, 2020, p. 53). The police were particularly associated with human rights violations. Law enforcement has been well regarded since the return to democracy in 1990, with a reputation for professionalism and integrity (Bonner, 2018). However, to their involvement in human rights violations to repress the protests were added several accusations of embezzlement, frame-ups, and cover-ups in recent years (Viollier Bonvin et al., 2019; Barbosa dos Santos et al., 2021; Montt et al., 2021). Thus, the Carabineros’ reputation for community policing has been put in jeopardy due to their involvement in human rights abuses and excessive use of force against civilian populations (Malone and Dammert, 2021, p. 430). The presence on the streets and repression by the armed forces also recalled Chileans of the military’s role during the dictatorship, bringing back dark memories to many citizens (Aliaga, 2021).

Given the unprecedented but contested scale of human rights violations during the Chilean social outburst, the main goal of this study is to analyze the determinants of perceptions of human rights using evidence from public opinion. By analyzing this case we aim to contribute to several strands of research. First, we seek to add to the psychological corpus on human rights attitudes (Cohrs et al., 2007; Crowson and DeBacker, 2008; McFarland, 2010; Krause, 2022), which has identified several individual-level and attitudinal predictors, by gauging the explanatory power of important attitudinal and contextual variables. Second, while the Latin American research on crime and policing has shed light on public support of human rights restrictions (Dammert and Malone, 2006; Cruz, 2009; Uang, 2013; Pion-Berlin and Carreras, 2017; Visconti, 2020; Bonner and Dammert, 2022), we also examine the potential impact of protests on human rights perceptions. Finally, by studying human rights perceptions in a recent event, this article seeks to contribute to the Chilean social science research on human rights, which has focused primarily on violations during the dictatorship of Augusto Pinochet (1973–1990) (Roniger and Sznajder, 1999; Oteíza, 2009; Collins, 2010; Cárdenas et al., 2013; Heinz and Frühling, 2021; Rojas, 2021).

We use the December 2019 CEP survey, a nationally representative survey fielded during the outburst, to evaluate four context-specific determinants. Specifically, we use (1) participation in the protests, (2) media use for political information, (3) fear of crime, and (4) geographic proximity to violent protests in ordered logistic regressions of perceptions of the frequency with which the police and armed forces violated human rights during the social outburst. We find that all four variables are correlated with the opinion that the police violated human rights very frequently, while only (3) and (4) are significantly associated with saying the military violated human rights very frequently. However, the association between fear of crime counters theoretical expectations, as increased worry about victimization is positively correlated with more critical views of security forces.

The rest of the article is structured as follows. The second section presents and discusses the relevant literature on human rights attitudes and perceptions, both generally and in Latin America, while the third section presents our hypotheses based on the literature. The fourth section describes our research design and methodology, including the way we operationalize the variables to test our hypotheses. The fifth section lays out the results of eight ordered logistic regressions of the CEP data. The sixth section presents our conclusions.

## 2. Theorizing about human rights perceptions

The study of attitudes toward human rights has made important strides in recent decades (McFarland, 2015). Several works have conceptualized and elaborated distinct measures of human rights attitudes, which have also been empirically evaluated. While some works have focused on normatively positive dispositions toward human rights (McFarland and Mathews, 2005) such as commitment to enforcement of human rights (Pratto et al., 1994, p. 750; Fetchenhauer and Bierhoff, 2004), and even behavior supporting human rights (Cohrs et al., 2007; Sommer and Stellmacher, 2009), a strand of this research has also delved into attitudes toward human rights restrictions. This distinction is important because, while a general support for human rights may become a valence issue (Stokes, 1963, p. 373), people may be open to ignoring human rights for specific groups or under certain circumstances. As Sommer and Stellmacher (2009, p. 65) state, “the realization of human rights is unanimously regarded as very important. At the same time, concrete knowledge of what human rights are is very limited and imprecise.” Restrictions in the United States literature range, for instance, from limiting Communists’ right to vote to denying constitutional rights to disloyal citizens (McFarland and Mathews, 2005).

Some works have also studied human rights restrictions under situations of heightened external threat. Crowson et al. (2006), for example, find that some people are willing to constrain human rights in the context of the War on Terror in the United States, while experimental evidence shows that support for torture increases when survey respondents are told detainees are Arab terrorist suspects (Conrad et al., 2018). Meanwhile, using data from 19 countries, Kull et al. (2008) find that, although people in most countries are against the use of torture, countries where the majority of respondents are open to the government torturing potential terrorists for information had experienced terrorist attacks recently. Likewise, in Latin America, crime victimization is positively correlated with approving of the police using torture to obtain information from criminals (Krause, 2022). There are fewer works, however, on attitudes toward limiting human rights in the context of widespread social mobilization and civil turmoil, and with Global South samples (Carriere, 2019, p. 20).^1^

In Chile, human rights violations during the outburst remain a hotly debated issue. The government initially denied human rights violations during the demonstrations before the Inter-American Human Rights Court (El Mostrador, 2019). The police, in turn, presented its own account of the facts, and also denied human rights abuses (El Mostrador, 2021). Meanwhile, the director of the National Institute of Human Rights Institute, the public organization in charge of promoting and defending human rights, claimed that the violations were “not systematic,” meaning that the Chilean state did not commit human rights abuses according to a specific plan or policy (T13, 2019). Regarding these abuses, the director also claimed that “there are no rights without duties,” causing an uproar both within the Institute and among leftist politicians (Pressacco and Castillo, 2022, pp. 95–96). José Antonio Kast, the ultraright presidential runner-up, has gone so far as to say that mass human rights violation did not exist, and that the political left promoted urban violence with its accusations (Kast, 2021).

The literature has also identified a few consistent predictors of human rights support. Generalized prejudice (Allport, 1979) for example, “would seem anti-thetical to support for human rights” (McFarland, 2015, p. 17). The two roots of this generalized rejection of outgroups, in turn, are right wing authoritarianism and social dominance orientation, which have been found to be consistently and negatively correlated with human rights support. The same association has been found in Latin America, controlling for country-level covariates, and is larger than that of more traditional predictors like crime victimization (Krause, 2022). However, the literature on human rights attitude formation has, with some exceptions (Barton et al., 2017) been restricted to attitudinal, individual-level predictors, while works in political science have primarily used countries and country-years as units of analysis (Miller, 2011; Joshi et al., 2019; Krause, 2022). There is, therefore, ample room for research on local or subnational level predictors of human rights perceptions, which are key for political attitude formation (Hiskey and Bowler, 2005).

In the specialized Latin Americanist literature, human rights have been extensively studied with regards to policing and crime. The region has historically suffered from high levels of crime and violence (Soares and Naritomi, 2010), while fear of crime is high and policing and crime prevention are very salient political issues (Dammert and Malone, 2006; Uang, 2013). Thus, although they only partially explain human rights attitudes (Krause, 2022), both crime victimization and fear of crime are important predictors of support for iron-fist, often illegal crime-reduction policies (Cruz, 2009; Visconti, 2020), which include using the military to quell crime (Pion-Berlin and Carreras, 2017), restricting civil liberties, and violating human rights (Cruz, 2000). As Krause (2022, p. 257) relates, for some Latin Americans “protecting the human rights of suspected criminals ties the hands of the police and the courts. This rhetoric revokes the universality of human rights by privileging public security over the protection of the human rights of all citizens.”

Finally, in Chile, most studies on human rights deal with the memory of abuses committed by the Pinochet dictatorship. Collins (2010), finds lasting divisions in attitudes toward human rights violations during the dictatorship, while Rojas (2021) identifies the correlates of indifference toward human rights violations in the same period. Meanwhile, fewer publications address attitudes toward human rights since the return to democracy. It is worth noting, for instance that the INDH has fielded fives waves of its National Human Rights Survey since 2011, describing several measures of human rights attitudes (INDH, 2020, p. 2). Other exceptions have to do with foreign policy: some surveys have described preferences for policies regarding human rights violations in other countries (Morandé et al., 2009), and the level of support for democracy and human rights promotion internatioally (Athena Lab, 2022, p. 10). We were able to find one study (Aguilera and Badilla Rajevic, 2022) focusing on humans rights opinions during the Chilean outburst, but it focused on the vandalization of dictatorship-era human rights memorials during the social unrest in 2019.

## 3. Determinants of perceptions of human rights violations

While several factors may influence perceptions about human rights violations during episodes of social unrest, we focus on four key behavioral, attitudinal, and geographic determinants. We focus on these determinants because they speak to the way social and political contexts shape political attitudes and perceptions. As Gibson and Gouws (2001, p. 1071) explain with regards to the causes of political tolerance,

Instead of *recalling* opinions, many respondents are actually *creating* opinions, deriving them from the particular values stimulated by the question. Real politics involves judgment; it typically involves figuring out how incidents in the political environment connect with attitudes and values and, more important, how conflicts among competing values get resolved.

In other words, individual characteristics, attitudes, and behaviors interact with the national, local and temporal context, which should, therefore, be considered.

The first two determinants deal with behaviors during the 2019 Chilean social outburst; the next gauges the importance of attitudes related to crime; the last one hypothesizes the impact of physical proximity to violent protests on perceptions about the human rights violation committed by security forces. The variables are chosen because they may have specific associations with human rights attitudes in the Latin American and Chilean context, the subnational and local circumstances, and the specific period of the social outburst.

### 3.1. Protest participation

A key determinant of perceptions of human rights violations during civil unrest is participation in the unrest itself. Indeed, the biographic effects of protests participation are well attested in the social movement literature (Sherkat and Blocker, 1997; Giugni, 2004). Scholars have found, for example, that demonstrating increases political engagement through voting (Galais, 2014) and subsequent participation (Bursztyn et al., 2021), and that engaging in aggressive political behavior reduces regime support (Finkel, 1987). Importantly, taking part in demonstrations causes participants to align their perceptions with the social movement’s frames and demands, unifying people with different motivations and preferences, and framing their discontent into a small number of claims that reinforce existing attitudes and prompts attitude change on issues central to the protests (Pop-Eleches et al., 2022, p. 626). Thus, to the extent that protests during the outburst also became against police brutality (Badilla Rajevic, 2020, p. 286), participants also became more critical of human rights violations committed by security forces.

In a similar vein, participation in mobilizations may give demonstrators first-hand experience of repression, especially during the “[v]iolent spirals of repression-protest-repression developed during the Chilean uprising” (Somma, 2021, p. 587). In this regard, studies in policing and criminology have found that procedural injustice shapes to a great extent attitudes and behaviors during protest cycles. Perceptions of unjust treatment by security forces are associated with decreased compliance with the police (Perry, 2020), increased justification of violence against law enforcement (Maguire et al., 2018a,b), and violent actions against security forces (Tyler et al., 2018). Repression, therefore, may decrease the legitimacy of security forces in the eyes of demonstrators (Bonner and Dammert, 2022), increasing perceptions of human rights violations. Meanwhile, evidence from the October outburst in Chile shows that perceptions of procedural injustice toward demonstrators are negatively correlated with justification of violence by the police against protesters (Gerber et al., 2023).

*Hypothesis 1*: Participating in demonstrations during the October outburst is positively associated with the perception that security forces violated human rights.

### 3.2. Media use

Media use for political information can shape perceptions of both protest participants and nonparticipants. Media consumption can have important effects on social and political perceptions, and they can contribute to mental models about the appropriateness and morality of certain actions (Mares and Stephenson, 2017, p. 2). The relationship between human rights attitudes and media use is nevertheless understudied (Cohrs et al., 2007, p. 462). Experimental research shows, for example that immersive journalism, which aims at increasing empathy, has a positive effect on human rights commitment (Bujić et al., 2020).

The effect of media use during the outburst on human rights perceptions may depend on the specific characteristics of the media ecosystem in Chile. The levels of media ownership concentration are high by Latin American standards (Núñez-Mussa, 2021), with traditional print and broadcast media outlets tightly associated with the economic elite (Valenzuela and Arriagada, 2011). Although trust in the media has decreased in recent years (Newman et al., 2021), these outlets have an important agenda setting power: public opinion is highly responsive to news coverage – but the influence does not go the other way around (Valenzuela and Arriagada, 2011). The Chilean traditional media has been characterized as politically conservative (Gronemeyer et al., 2021), highlighting violence during previous episodes of mass protest (Kubal and Fisher, 2016, p. 230), and rejecting social movements’ demands (Cabalin, 2014). The evidence shows, for example, that *Radio Biobío*—the country’s most trusted news source—“replicated the patterns that usually delegitimize the protest, as they focused on the violent acts and the depiction of protesters as deviant from the status quo” (Proust and Saldaña, 2022, p. 25). Meanwhile, statements by supporters of protest policing in *El Mercurio* and *La Tercera*—the country’s most important newspapers—“justified high levels of police violence and opposed critiques of human rights abuses” (Bonner and Dammert, 2022, p. 641).

*Hypothesis 2a*: Using more media for political information during the October outburst is negatively associated with the perception that security forces violated human rights.

However, the media landscape experienced relevant transformations during the outburst. While news sharing in social media of traditional outlets experienced a temporary increase during the outburst, public opinion became critical of the role played by these actors (Grassau et al., 2019). Meanwhile, alternative media sources experienced a more lasting shock. This boost helped alternative, online outlets to counter mainstream narratives, causing public opinion “to question traditional media and its coverage of the riots, social unrest, and police repression and human rights abuses” (Luna et al., 2022, p. 2). In some traditional outlets, the scope of disruption and repression caused journalists to question more detached approaches to the production of news content, leading some to adopt an “epistemic mode where journalists validate the decision to take a position in the conflict, which in the uprising was a commitment to stories of police abuse and human rights violations” (Orchard and Fergnani, 2022, p. 12).

*Hypothesis 2b*: Using more media for political information during the October outburst is positively associated with the perception that security forces violated human rights.

### 3.3. Fear of crime

The psychological literature has delved on the way perceived threats shape social and political attitudes. Broadly, threats can be either realistic – against physical integrity – or symbolic – against values and morals (Carriere, 2019, pp. 11–12). Critically, support for the rights of out-groups depends on their level of perceived threat (Verkuyten, 2009; Abrams et al., 2015). As Crowson et al. (2006, p. 737) explain, salient threat information can prompt individuals to adopt a defensive stance, potentially influencing sociopolitical outcomes in combination with individual factors. Several works have found, for example, that terrorism affects attitudes toward security (Brouard et al., 2018), and that perceived threat of terrorism increases support for torture (Conrad et al., 2018).

As mentioned above, fear of crime is a widespread, perceived realistic threat in Latin American societies—and Chile is not an exception (Dammert and Malone, 2003). The country has high levels of fear of crime with relatively low levels of crime victimization—a paradox that been explained in terms of economic anxiety (Dammert and Malone, 2003; Singer et al., 2020) and media portrayals of crime (Browne and Tomicic, 2007; Scherman and Etchegaray, 2013). Thus, the perception of crime is biased because as “the media disproportionately focuses on violent crime (epitomized by the media adage, ‘if it bleeds, it leads’) the public becomes more fearful of violent crime, even when objective crime rates decrease” (Dammert and Malone, 2003, 89–90). Critically, fear of crime “may push citizens toward harsher attitudes toward potential criminals based on the idea that restricting the rights of alleged criminals may make their neighborhoods safer” (Krause, 2022, p. 259) Thus, to the extent that protest is criminalized by the authorities (Doran, 2017), individuals who are very afraid of crime may perceive that human rights violations occurred less often during the outburst.

*Hypothesis 3*: Increased fear of crime during the October outburst is negatively associated with the perception that security forces violated human rights.

### 3.4. Protest proximity

The political science and sociological literatures have found that physical proximity to social mobilizations can shape political attitudes and perceptions about protest demands, actors, and targets in various contexts (Wallace et al., 2014; Mazumder, 2018; Muñoz and Anduiza, 2019; Ketchley and El-Rayyes, 2021), and Chile is not an exception (Disi Pavlic, 2021). Spatial and temporal proximity to student mobilizations in the country, for example, is positively correlated with political interest and the perception that government critics have a right to protest peacefully.

From a psychological standpoint, proximity to violence in protests may decrease support for the mobilizations and increase support for state repression because violence in protests may also be perceived as a threat, with the potential to cause bodily harm and transgress values. The use of violent tactics decreases identification with protest participants as they are perceived as less reasonable (Simpson et al., 2018), while threat of harm by demonstrators has been found to increase support for repression (Edwards and Arnon, 2021). For example, in the context of the protests in 2020 sparked by the killing of George Floyd in the United States, “when protesters were delaying traffic, carrying firearms, or behaving unlawfully (damaging property and/or assaulting citizens), fear of the protesters increased, which in turn increased support for police repression” (Metcalfe and Pickett, 2022).

*Hypothesis 4*: Residing in locations with more violent protests during the October outburst is negatively associated with the perception that security forces violated human rights.

## 4. Methods

We leverage observational data from the 84^th^ *Centro de Estudios Públicos* (CEP) Survey (CEP, 2020) to evaluate our hypotheses. This data gives us the unique chance us to analyze perceptions of human rights violations when domestic and international actors accused the Chilean state not just of limiting civil liberties but also of using unprecedented levels of repression to quell social unrest.

### 4.1. Participants

A total of 1,496 individuals participated in the 84^th^ CEP survey. Respondents’ age ranged from 18 to 99 (M = 44.2, SD = 17.5); 48.3% were male and 51.7% female; their reported levels of education were less than secondary education (30.5%), complete secondary (33.3%), incomplete higher education (10.1%), complete college (23.1%), and graduate education (3%); 87.% lived in urban areas and 12.4% were rural residents; and their socioeconomic levels were ABC1 (highest, 5.6%), C2 (12.9%), C3 (52%), D (28.5%), and E (lowest, 1%). The survey was administered to a sample of individuals that is representative of national adult population, using a multi-stage (block/household/adults) probability sampling design, stratified by first-level administrative divisions (16 regions) and geographic zones (urban/rural). They survey uses sampling weights based on the 2017 census to account for nonresponse and oversampling of certain demographics. Respondents agreed to participate voluntarily and were told they could withdraw at any moment and decline to answer any question.

### 4.2. Procedure

**Figure 1:**
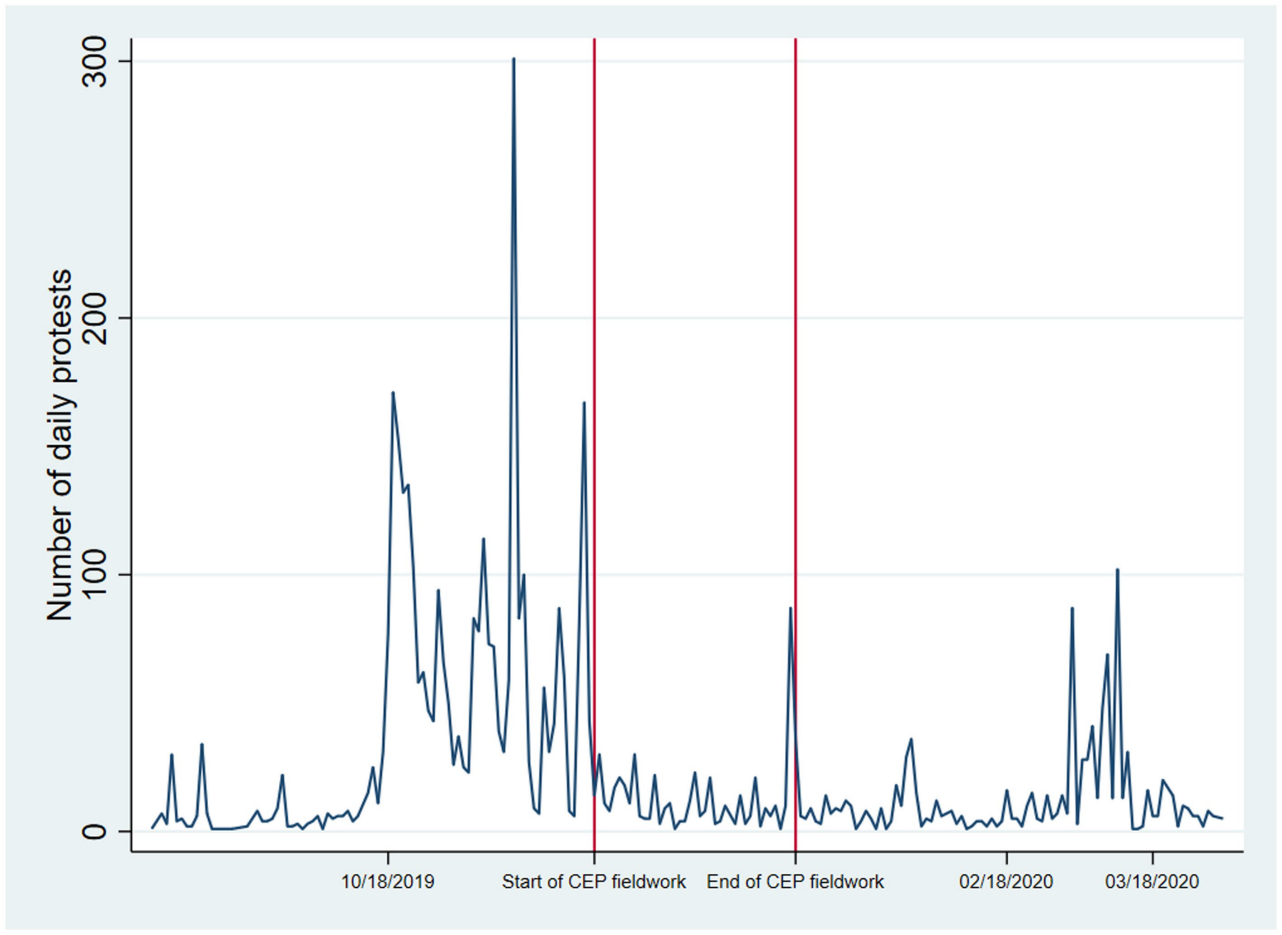
Frequency of protests during the 2019 Chilean unrest and CEP fieldwork dates.

Fieldwork and data collection were conducted between November 28, 2019, and January 6, 2020. As Figure 1 shows, data from the Centre for Social Conflict and Cohesion Studies (COES, 2020) shows that the survey’s fieldwork took place right before and during some of the most contentious moments of the Chilean social outburst.

The sample considered 2,128 participants, which resulted in 1,496 effective interviews, with a response rate of 71%. The sample covered 98% of the target population. Respondents answered a questionnaire containing questions about their political, economic, and social attitudes and predispositions. Trained pollsters used tablets to administer the questionnaire through the Computer Assisted Personal Interview (CAPI) system. The survey has a sampling error of plus or minus 3% points at the 95% confidence level.

### 4.3. Measurement

The study uses two dependent variables, as well as six independent variables. One of these predictors varies by district and its values are, therefore, shared by respondents in the same location, while the other five vary by individual. The article also uses 10 other predictors that the literature has found to be correlated with human rights attitudes.

#### 4.3.1. Dependent variables

We take advantage of two items specifically designed for this wave of the CEP survey to operationalize our dependent variable. Respondents were asked to assess, using five-point Likert scales, the level of frequency that *Carabineros* and the armed forces committed human rights violations during the social outburst.^2^ The categories ranged from “Never” to “Very Frequently.”

#### 4.3.2. Independent variables

The hypotheses presented above are evaluated using five independent predictors. The first one is used to assess the association between perceptions of human rights violations and participation in protests during the social outburst (Hypothesis 1). An item about participation in demonstrations (*marchas*) during the 2019 crisis was dichotomized to measure potential exposure to state repression through protest participation.^3^

Hypotheses 2a and 2b are tested using the next three independent covariates. These predictors are measures of respondents’ use of different types of media. We leverage data from three questions about three types of behavior: watching political television shows; reading political news; and following political issues on social media platforms “such as Facebook and Twitter.” The items are measured using ordered, three-point scales (“frequently,” “sometimes,” “never”). The first media variable combines all these items to create a scale of political media use frequency (*α* = 0.8, *ω* = 0.8). The second media variable is a scale of the first two (*α* = 0.75), which refer to traditional mass media and may have a different effect on political behavior and attitudes than newer, online platforms (Navia and Ulriksen, 2017).^4^ The last one is the social media item. The variable combining the three items is used separately from the other two media variables in the regressions models below.

The fifth independent variable is used to assess the correlation between fear of crime and human rights perceptions (Hypothesis 3). We use a question from the CEP survey asking respondents how worried they were about becoming victims of violent crime.^5^ The categories in the 11-point scale range from “not worried at all” (0) to “very worried” (10).

Finally, the sixth independent covariate considers the potential effect of the local protest context (Hypothesis 4). Thus, we use geocoded data from COES (2020) to count, for each of Chile’s 345 *comunas* (first-level administrative districts), the number of violent protests in the district itself and in a 10-kilometer buffer zone from the district limits between October 18 and December 31, 2019.^6^ The CEP sample, with its 117 districts, includes respondents in the full range this variable, from zero nearby protests for 234 respondents in 26 *comunas* to 109 violent events for 19 respondents in the district of San Miguel.

#### 4.3.3. Other predictors

Ten theoretically-informed predictors variables are incorporated into the analyses. The first five are important attitudinal predictors, according to the literature, of opinions about human rights. The first one is political ideology, which is used as a categorical variable with seven categories: Right (4.1% of valid answers), Center-Right (3.8%), Center (6.3%), Center-Left (6.1%), Left (10%), Independent (2.6%) and None (67.2%). Ideology is included because the psychological literature on human rights attitudes points out that ideology, and particularly conservative and right-wing positions are negatively associated with support for human rights (Moghaddam and Vuksanovic, 1990; Diaz-Veizades et al., 1995; Crowson, 2004; Crowson and DeBacker, 2008). While there are fewer studies on the Chilean case, the 2020 National Human Rights Survey finds that a higher percentage of people with a Right-wing ideology (though still the minority) consider that sometimes it is necessary, although not desirable, to violate some people’s human rights (INDH, 2020, p. 38).

The second and third of these variables deal with trust. Institutional trust is linked with specific political support (Easton, 1975) and, in the case of the police, it “condenses a range of complex and inter-related judgements concerning the trustworthiness of the police” (Lee et al., 2015, p. 241). Evidence from Mexico shows that human rights perceptions are significantly correlated with trust in security forces (Barton et al., 2017). In Chile, the perception that the armed forces respect human rights is positively correlated with trust in both the military and the police (Riffo et al., 2019, pp. 95–96). Additionally, it is important to control for trust in the security forces because trusting them is negatively correlated with fear of crime (Dammert and Malone, 2002, p. 297; Malone and Dammert, 2021, p. 427). Trust in these two actors is measured through 4-point Likert scales ranging from “A lot of trust” to “No trust” (M = 3.3, SD = 0.8, for the police, M = 3.1, SD = 0.9 for the armed forces).

Preferences for security, understood more broadly than just protection from crime, are also included because they have also been identified as predictors of human rights attitudes, with increased security concerns being correlated with preferences for restricting human rights (Cohrs et al., 2007). We use an item using a scale of one to ten measuring reactions to the statement “Democracies aspire to have public and private liberties and public order and safety. In your case, which value do attach more importance to?,” with one being “public and private liberties” and ten “public order and safety” (M = 6.2, SD = 2.8).

The last attitudinal variable assesses the association between authoritarianism and human rights perceptions. Right-wing authoritarianism (Altemeyer, 1998), for example, has been found to be positively correlated with preferences for restricting of human rights during crisis situations (Crowson and DeBacker, 2008), while authoritarianism in general is indirectly associated with lesser commitment with human rights (McFarland, 2010, p. 1755). The CEP survey includes four questions^7^ that we use to create a scale of authoritarianism (*α* = 0.8, *ω* = 0.9).

The other seven variables are sociodemographic covariates, all of which shape public opinion and human rights attitudes, according to the relevant literature. The fifth predictor is respondents’ socioeconomic level. In Latin America, poorer citizens are more critical of human rights protections (Gao et al., 2019, p. 3), while in Chile lower-income people tend to hold more negative perceptions of the state of human rights protections in the country (INDH, 2020, p. 48). Respondents’ location in urban or rural areas is the sixth predictor. It has been found, for example, that knowledge about human rights is higher among urban dwellers (Koo et al., 2015). Religion is added as a seventh predictor variable because some denominations have been found to be correlated with decreased support for human rights (Narvaez et al., 1999; Urueña, 2019). We categorize the sample into four categories: Catholic, Protestant, Other, and None. The eighth predictor is education. Similar to the poor, people with fewer years for formal education tend to be more critical of the enforcement of human rights in Latin America (Gao et al., 2019, p. 4). Gender has also been found to be correlated with attitudes toward human rights, with females being less supportive of restrictions on human rights (Crowson and DeBacker, 2008, p. 304), and placing more importance in Chile on living in a country that respects human rights (INDH, 2020, p. 33). The last predictor is age, which has been found to be correlated with human right attitudes in different contexts. For example, older Americans are less willing to spend more money on products that respect worker’s human rights (Hertel et al., 2009, p. 456), while in Chile older age is associated with the perception that there is little respect for human rights (INDH, 2020, p. 32). These covariates are used in the regressions detailed below.

### 4.4. Statistical analysis

After presenting the frequency distributions of the dependent variables, non-parametric bivariate analyses are used to assess the relationship between perceptions of human rights violations and protest participation, media use, fear of crime, and protest proximity. The dependent variables are measured using 5-point Likert scales and have skewed values, while Shapiro–Wilk [W(1,425) = 0.98859; *p* < 0.0001 for the police; W(1,400) = 91.15; *p* < 0.0001 for the military] and skewness and kurtosis tests [adjusted $\chi^{2}$ (1,425) = 91.15; *p* < 0.0001 for the police; adjusted $\chi^{2}$ (1,400) = 77.9; *p* < 0.0001 for the military] show that the dependent variables are not normally distributed, so tests like the Pearson and point-biserial correlation coefficients would not be appropriate. Instead, the bivariate analyses use Fisher’s exact test and Spearman’s rank correlation coefficient to evaluate the hypotheses (see Table 1).

**Table 1:** Descriptive statistics of dependent and independent variables (CEP, 2020).

	Perception of frequency of human rights violations		Demonstrated during the unrest	Media use frequency (all)	Traditional media use frequency	Social media use frequency	Fear of crime	Nearby violent protest
Statistic	Police	Military						
*N*	1,427	1,402	1,456	1,494	1,494	1,463	1,488	1,496
Mean	2.85	2.48	0.25	1.57	1.62	1.47	8.26	33.07
Min	0	0	0	1	1	1	0	0
Max	4	4	1	3	3	3	10	109
SD	1.11	1.22	0.43	0.57	0.61	0.66	2.49	35.12
Skewness	−0.75	−0.44	1.18	0.78	0.67	1.08	−1.76	0.74
Kurtosis	2.94	2.36	2.38	2.78	2.56	2.96	5.69	2.07
25th percentile	2	2	0	1	1	1	7	2
median	3	3	0	1.33	1.5	1	9	20
75th percentile	4	4	0	2	2	2	10	62

The determinants of perceptions of human rights violation are also tested using multivariate regressions. These models use, in addition to the independent variables, the other theoretically relevant predictors described in the Methods section. Ordered logistic models are used because they are appropriate when dependent variables are ordinal and have a limited range of values (Cameron and Trivedi, 2013). However, to assess the robustness of the results, linear regressions are also used. The models also include robust standard errors, and the survey population weights. Finally, predicted values are used to illustrate some of the results. The analysis were carried in Stata 17 (StataCorp, 2021).^9^

## 5. Results

A series of descriptive statistics were performed for the dependent and independent variables. These statistics are shown in Table 1. Most respondents in the CEP survey answered that the country’s security forces committed human rights violations at least sometimes during the social outburst. In the case of the police the share is higher, with more than one third of the sample stating that Carabineros violated human rights very frequently. Meanwhile, the most recurrent answer for the military is “sometimes,” although almost half of the sample stated that they violated human rights frequently or very frequently. The answers in both cases are, therefore, skewed toward higher frequencies of human rights violations.

Next, we analyze the bivariate relationships between the dependent and independent variables using non-parametric tests. In the case of protest participation, since it is binary, we use the chi-square exact test to assess its association with the dependent variables. The results show that demonstrating during the unrest is positively associated with perceptions that the police ( $\chi^{2}$ (4) = 119.2157; *p* < 0.0001) and the military violated human rights very frequently ( $\chi^{2}$ (4) = 83.8252; *p* < 0.0001). The results, therefore, support Hypothesis 1.

With the rest of the variables, we use the Spearman’s rank coefficient because it is appropriate for ordinal covariates. More frequent uses of general [ $r_{s}$ (1,424) = 0.0898; *p* < 0.001], traditional [ $r_{s}$ (1,424) = 0.0458; *p* < 0.1], and social media [ $r_{s}$ (1,398) = 0.1460; *p* < 0.0001] are positively correlated with the police variable; the frequencies of using general [ $r_{s}$ (1,399) = 0.0639; *p* < 0.05] and social media [ $r_{s}$ (1,424) = 0.1176; *p* < 0.0001] are positively correlated with the armed forces variable. Thus, correlations support Hypothesis 2b about the positive association between media use and perceptions of more frequent human violations. Fear of crime is also significantly correlated with human rights perceptions, though not as expected. In the case of the police, the correlation is positive [ $r_{s}$ (1,419) = 0.0692; *p* < 0.05]. The observed inverse directionality of the relationship contradicts the anticipated outcome stated in Hypothesis 3. Finally, the results suggest there is a significant bivariate relationship between the number of nearby violent protests and human rights violations perceptions. The correlation is positive for the police [ $r_{s}$ (1,425) = 0.0516; *p* < 0.1] and military variables [ $r_{s}$ (1,399) = 0.0554; *p* < 0.05]. This lends support to Hypothesis 4.

**Figure 2:**
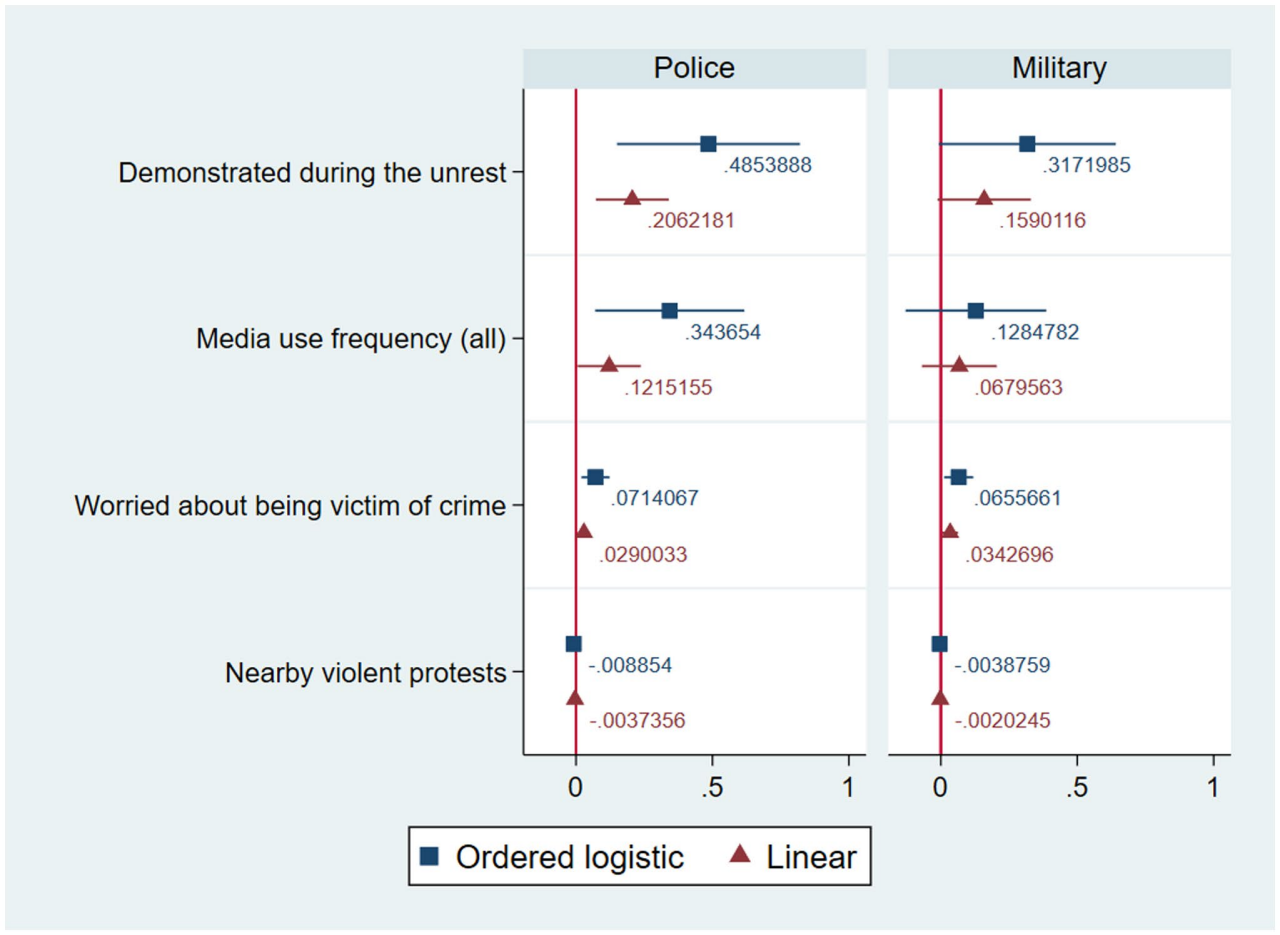
Regressions of perceptions of frequency of human rights violations by the police and military during the October crisis (all media), with 95% confidence intervals.

We carried out eight multivariate regressions. Four use ordered logistic models, and four use ordered least squares models. Half of the models use the media scale variable (TV, reading, and social media), and the other half use the traditional media scale (TV, reading) and social media variables.^10^ Figure 2 plots the coefficients for the two outcomes in ordered logistic and linear regressions using the general social media variable. In these models, demonstrating during the social outburst is positively correlated with stating that the police violated human rights very frequently during the October crisis (ordered logit *β* = 0.48; *p* < 0.01; linear *β* = 0.21; <0.01). Meanwhile, the association with protest participation is not statistically significant for the military. Hypothesis 1, therefore, is supported but only for the police. Likewise, media use is positively correlated with answering that the police violated human rights very frequently (ordered logit *β* = 0.34; *p* < 0.05; linear *β* = 0.12; <0.05) while the effect is null for the military. Hypothesis 2b, then, is supported only with regards to the police, while Hypothesis 2a is refuted. Hypothesis 3, meanwhile, is fully rejected: worry about crime is positively associated with stating that human rights were violated very frequently by the police (ordered logit *β* = 0.71; *p* < 0.01; linear *β* = 0.03; <0.05), and by the military (ordered logit *β* = 0.07; *p* < 0.05; linear *β* = 0.03; <0.05). Thus, the correlations with fear of crime are statistically significant but their direction goes against theoretical expectations. Finally, Hypothesis 4 is partially supported on both accounts. Nearby violent protests are negatively correlated with saying that the police violated human rights frequently (ordered logit *β* = −0.01; *p* < 0.001; linear *β* = −0.004; <0.001), and the opinion that the military did as well (ordered logit *β* = −0.04; *p* < 0.05).

**Figure 3:**
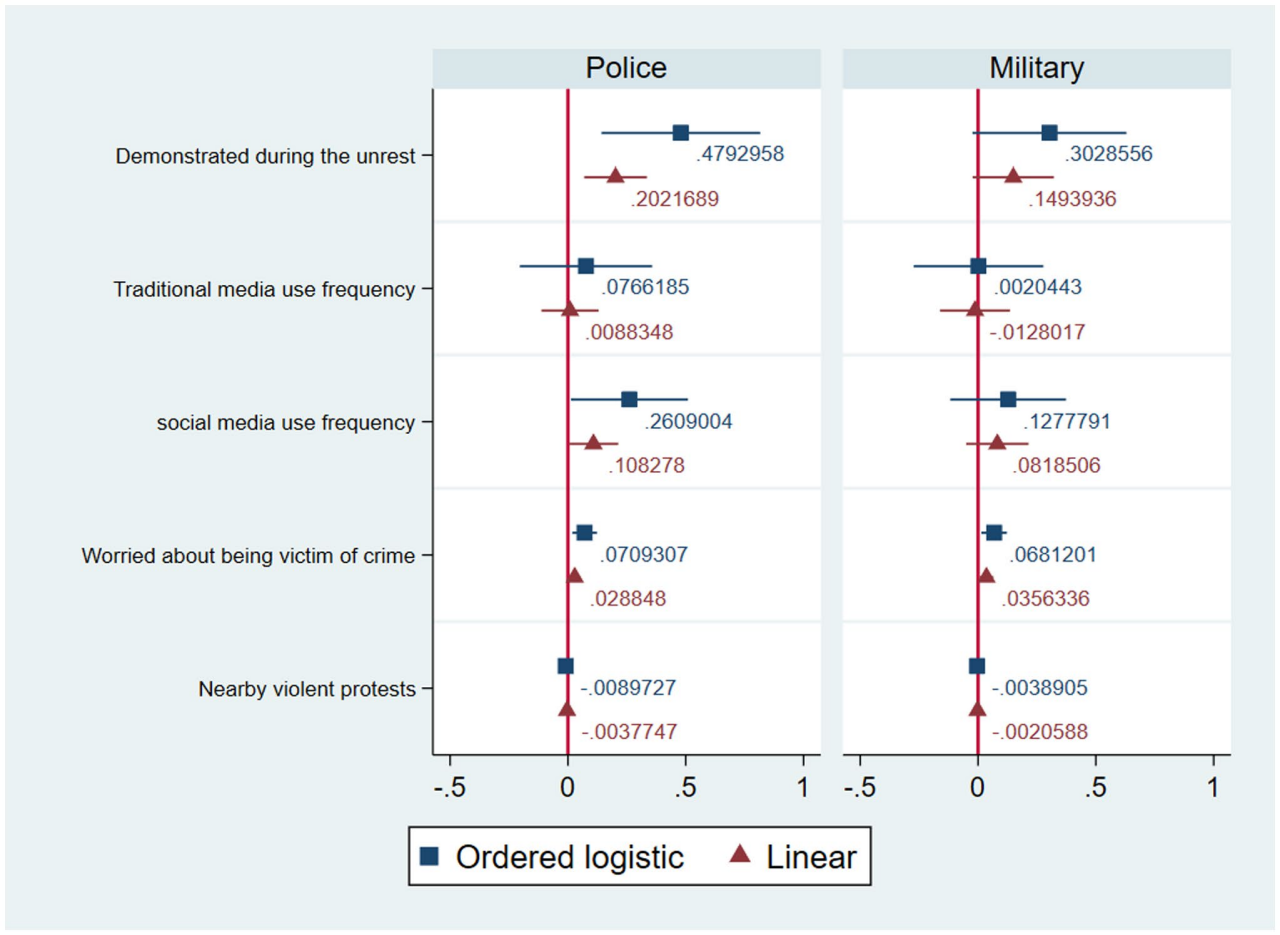
Regressions of perceptions of frequency of human rights violations by the police and military during the October crisis (traditional and social media), with 95% confidence intervals.

Figure 3 plots the same models as Figure 2 but using the traditional and social media variables as predictors instead of the general media one. In this case, the effects of demonstrating and fear of crime remain virtually the same, and the effect of nearby protests on perceptions of the military is now also statistically significant (linear *β* = −0.002; <0.05). With regards to the media variables, the effect of traditional media use is null across the models, while the association with social media use is positive for perceptions about the police (ordered logit *β* = 0.26; *p* < 0.05; linear *β* = 0.11; *p* < 0.05) but not the military. This suggests that empirical support for Hypothesis 2b depends on the type of media used.

**Figure 4:**
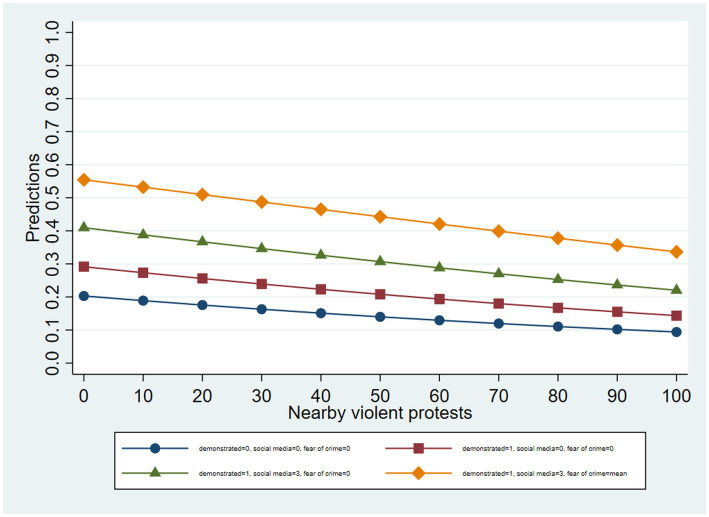
Adjusted predictions of stating that the police violated human rights “very frequently”.

To further illustrate the main results, we also estimate the adjusted predictions for selected values of the statistically significant independent variables in the models using ordered logistic regressions and the social media variable, while leaving the rest of the covariates at their mean values. Figure 4 shows the adjusted predictions of stating the police violated human rights very frequently along the range of observed values of nearby violent protests, and for specific values of the other predictors. Across the predictions, the probability of stating that the police violated human rights very frequently decreases as the number of violent protests in the respondents’ vicinity grows. Higher values of the other independent variables also increase this probability. For example, when respondents demonstrated during the crisis (value of 1); used social media frequently (3); and had a mean value in the fear of crime variable (8.2), the probability decreases from 55.4% with zero violent protests to 33.7% with 109 nearby events. This lends additional support to Hypotheses 1, 2b, and 4, while further rejecting Hypotheses 2a and 3.

**Figure 5:**
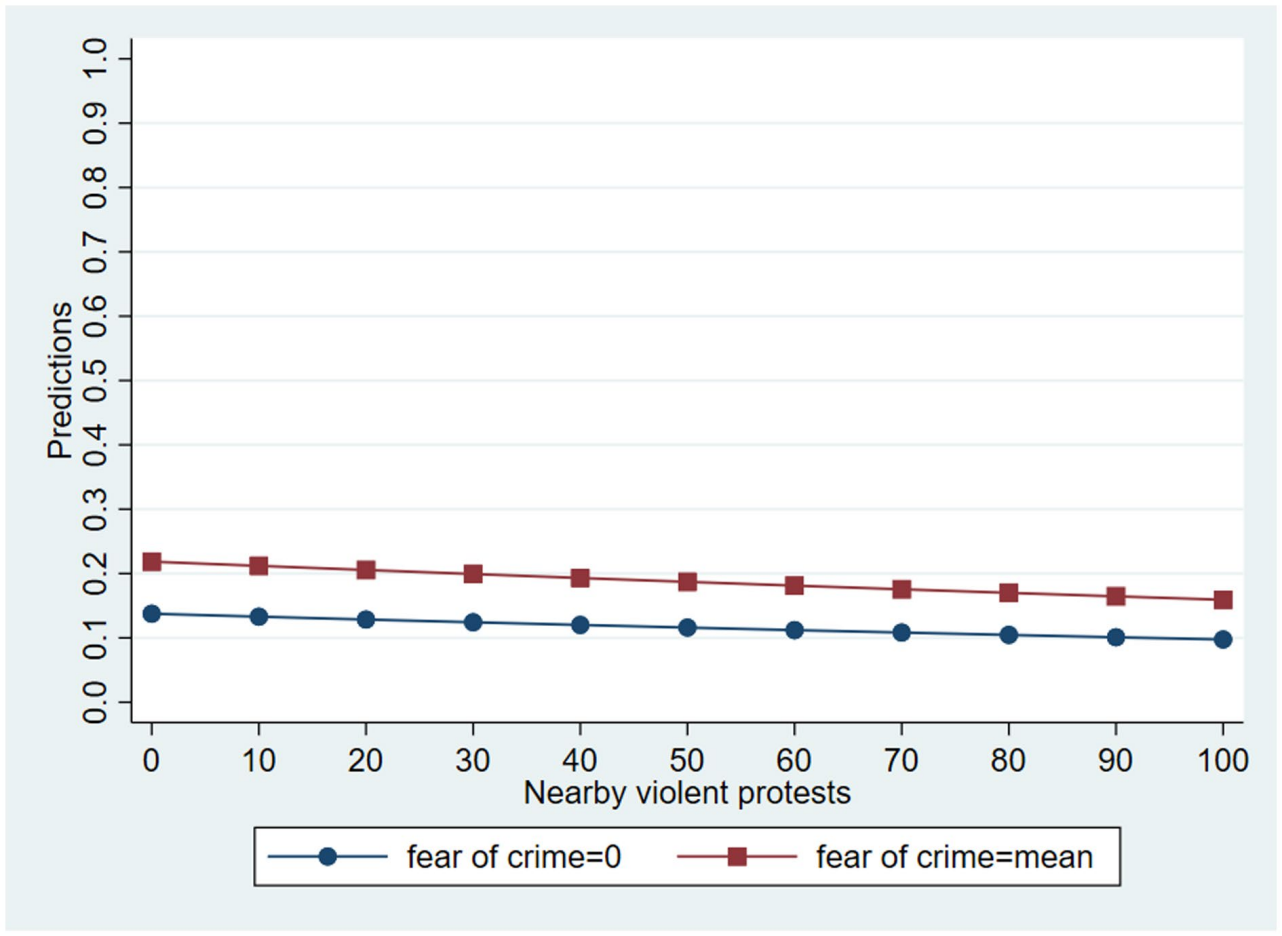
Adjusted predictions of stating that the armed forces violated human rights “very frequently”.

Figure 5 shows the adjusted predictions of the armed forces variable, in this case for protest participation and fear of crime (the only two statistically significant regressors). The probability of saying that the armed forces committed human rights violations very frequently goes up as nearby violent events increase, and it is higher for respondents who were more worried about becoming victims of crime. For example, when the fear of crime variable is at its mean, the probability declines from 21.9% with zero violent protests nearby to 15.9% with 109 violent events in the vicinity. These adjusted values support Hypothesis 4 and disprove Hypothesis 3.

## 6. Conclusion

The waves of protests that began on October 18, 2019, became the largest mobilizations in Chilean history. In addition to institutional and policy responses, the protests were met with unprecedented levels of repression and human rights violations, committed by security forces to suppress the unrest (Sehnbruch and Donoso, 2020). The extent of these violations remains a contentious issue to this day among political actors and in public opinion.

This work has sought to explain the predictors of perceptions of human rights violations during the October outburst by highlighting contextual and local variables. Drawing on the political science, sociology, and social psychology literatures, it hypothesizes that protest participation (Hypothesis 1), media use (Hypothesis 2a and 2b), fear of crime (Hypothesis 3), and spatial proximity to violent protests (Hypothesis 4) are correlated with the perception that Chilean security forces violated human rights frequently during the unrest. Thus, this article builds on but also goes beyond testing the generalizability of more traditional predictors such as right wing authoritarianism (Krause, 2022), education (Cruz, 2009; Gao et al., 2019), and ideology (INDH, 2020).

Bivariate and multivariate analyses of two indicators of perceptions of human rights violations in a nationally representative survey fielded during the outburst – one about the police and the other about the military—confirm three out of our five hypotheses. Participating in protests is positively correlated with the perception that the police (but not the armed forces) violated human rights frequently, partially confirming Hypothesis 1. It is worth noting that the regressions control for other predictors of demonstrating in Chile such as ideology, institutional trust, socioeconomic level, and age (Disi Pavlic and Mardones, 2019). Thus, our results contribute to the literature on the consequences of protest participation on political engagement (Thomas and Louis, 2013; Vestergren et al., 2017; Pop-Eleches et al., 2022), and to the analysis of the socio-cognitive processes operating during the Chilean outburst (Castro-Abril et al., 2021). Future research should check if the relationship between protesting and human rights perception is as pronounced in periods with less mobilization.

More media use for political information was found to be positively correlated with the perception that the police—but not that the military—committed human rights violations frequently. Thus, the results lend some support to Hypothesis 2b and refute Hypothesis 2a. They suggest that, although mainstream media outlets used frames emphasizing deviance and violence in protests and minimizing human rights violations (Chacón and Rivera, 2020; Bonner and Dammert, 2022; Proust and Saldaña, 2022) media consumption nevertheless generated a critical view of the way the police handled the protests. However, disaggregating media in traditional and social types showed that the association is with the latter. Thus, our results suggest that social media not only encouraged protest participation (Scherman and Rivera, 2021) but also impacted the political perceptions of the events that took place in the outburst. Future research could test these two hypothesis again but applying even more context-specific variables like using particular media outlets or framing typologies (Kozman, 2017) instead of media types.

It is worth noting that the protest participation and media variables only had a statistically significant association with the police variable but not the military. Additionally, the correlations on perceptions about the armed forces’ behavior are substantively smaller than those about police behavior for all the independent variables. The smaller, less significant effects on opinions about human rights violations by the military lends support to the argument that Latin Americans “lack a fundamental trust in law enforcement to do its job in a successful, transparent, and humane manner. By contrast, Latin American citizens place more trust in the armed forces as an institution capable of performing effectively, and in accordance with human rights standards and the rule of law” (Pion-Berlin and Carreras, 2017, p. 5).

Meanwhile, Hypothesis 3 is soundly rejected. Against theoretical expectations, increased fear of crime is *positively* associated with stating that both the police and the armed forces frequently violated human rights during the unrest. Thus, the results contradict the widespread argument that people are willing to do away with human rights protections in exchange for protection from crime (Sánchez, 2006; Ceobanu et al., 2011; Krause, 2022). The association between fear of crime and human rights perceptions may have less to do with perceived threats from crime (Carriere, 2019) and more with instrumental, outcome- or performance-based evaluations of security forces (Sunshine and Tyler, 2003; Murphy et al., 2008; Gau, 2010). The relationship may also be explained in terms of procedural fairness: people who perceive that the police does not curb crime may also be skeptical of their capacity to respect established protocols, procedures, or rights in their treatment of demonstrators (Liu and Wu, 2023). Thus, fear of crime does not give the police carte blanche to control other phenomena like protests—it may actually cause citizens to be more critical of the way the police carry out their duties. Finally, it may also be that most people—a even in a situation like the social outburst—make a distinction between and to dot equate ordinary crime with most types protest behavior (Sabucedo and Arce, 1991; Delfino and Zubieta, 2014).

Hypothesis 4 is supported, as the perception that security forces committed frequent human rights violations is positively correlated with the local occurrence of violent protest events during the unrest. The threat posed by violent protests, therefore, could increase justification or qualification of human rights restrictions (Metcalfe and Pickett, 2022). These results reaffirm the importance of within-country variation and subnational factors to understand political attitudes and human rights attitudes in particular (Barton et al., 2017; Crow, 2017), and contribute to our understanding of when public opinion justifies police violence (Jackson et al., 2013; Gerber and Jackson, 2017; Gerber et al., 2023). Other works could further explore the relationship between threats and support for human rights restrictions in the context of protest and civil unrest, which has usually emphasized other types of perceived threats (McLaren, 2003; Barth et al., 2015; Abrams et al., 2017). Finally, future research could explore the impacts other context-sensitive factors not included in this study, such as personal experience with human rights violations (Bautista et al., 2023) and prosecutions (Sikkink and Walling, 2007; Kim and Sikkink, 2010), and naming and shaming of one’s country by international organizations (Ausderan, 2014).

## Data availability statement

Publicly available datasets were analyzed in this study. This data can be found at: https://dataverse.harvard.edu/dataset.xhtml?persistentId=doi:10.7910/DVN/PKFJSY.

## Ethics statement

Ethical review and approval was not required for the study on human participants in accordance with the local legislation and institutional requirements. Written informed consent for participation was not required for this study in accordance with the national legislation and the institutional requirements.

## Author contributions

SC and RD contributed to the literature review, empirical analysis, and writing of the manuscript. All authors contributed to the article and approved the submitted version.

## Funding

This research was supported by ANID (Chile) through a FONDECYT Iniciación en Investigación grant [11190233], the Centre for Social Conflict and Cohesion Studies (COES) [ANID/FONDAP/15130009], and Dicyt-USACH.

## Conflict of interest

The authors declare that the research was conducted in the absence of any commercial or financial relationships that could be construed as a potential conflict of interest.

## Publisher’s note

All claims expressed in this article are solely those of the authors and do not necessarily represent those of their affiliated organizations, or those of the publisher, the editors and the reviewers. Any product that may be evaluated in this article, or claim that may be made by its manufacturer, is not guaranteed or endorsed by the publisher.

## Footnotes

^1^There are, however, works on the effect of protests on attitudes toward the right to protest (Andrews et al., 2016; Disi Pavlic, 2021), which itself a human right (Mead, 2010).

^2^Items E43 and E44 of the questionnaire read "How frequently did [*Carabineros*/the military] violate human rights during the crisis that began on October 2019?"

^3^Question E33.1 of the questionnaire reads "Did you participate in the demonstrations that began in October 2019, by attending a march?"

^4^I would like to thank an anonymous reviewer for suggesting this distinction.

^5^Question E22F: "In general terms, on a scale of 0 to 10, where 0 is not worried at all and 10 is very worried, how worried are you about the following situations? That someone in your family or you become victims of a violent crime (robbery, aggression)."

^6^Protests were coded as violent if the COES dataset registered at least one of the following tactics: arson; attacks against public and private property; looting; use of weapons and explosives; attacks against other demonstrators; clashes with counterdemonstrators; attacks against the police or armed forces; attacks against passers-by; taking hostages or kidnappings.

^7^Items E49A ("Instead of so much worrying about people’s rights, what this country needs is a firm government."), E49B ("What our country needs is a strong leader with the determination to take us on the right path."), E49C ("Obedience and respect for authority are the most important values that children should learn."), and E49D ("The true keys to a good life are obedience and discipline"), measured using a 5-point Likert scale of level of agreement with the statements.

^9^Replication files are available on the Harvard Dataverse (Disi Pavlic and Carrasco, 2023).

^10^For the full results with all the predictors see Table A1 for the logistic regressions (with odds ratios) and Table A2 for the linear regressions in the Appendix.
