# Supplementary material for: Predictors of perceptions of human rights violations during the Chilean social outburst of 2019
Source: Front Psychol. 2023 Apr 28;14:1133428. doi: 10.3389/fpsyg.2023.1133428 (PMC10210138; doi:10.3389/fpsyg.2023.1133428)
Supplement: Supplementary file 1 [file Data_Sheet_1.docx]

**Appendix for “Attitudes towards human rights violations during the 2019 Chilean social outburst”**

**Table A1.**

*Ordered logistic regressions of perceptions of frequency of human rights violations by the police and military during the October crisis.*

| Variables | Police | | Military | |
| --- | --- | --- | --- | --- |
| Demonstrated during the unrest | 1.625** | 1.615** | 1.373 | 1.354 |
|  | (0.278) | (0.277) | (0.227) | (0.226) |
| Media frequency use (all) | 1.410* |  | 1.137 |  |
|  | (0.197) |  | (0.150) |  |
| Traditional media frequency use |  | 1.080 |  | 1.002 |
|  |  | (0.155) |  | (0.141) |
| Social media frequency use |  | 1.298* |  | 1.136 |
|  |  | (0.165) |  | (0.142) |
| Worried about being victim of crime | 1.074** | 1.074** | 1.068* | 1.070* |
|  | (0.0282) | (0.0285) | (0.0290) | (0.0297) |
| Nearby violent protests | 0.991*** | 0.991*** | 0.996* | 0.996* |
|  | (0.00214) | (0.00216) | (0.00193) | (0.00194) |
| Ideology (reference=Right) |  |  |  |  |
| *Center Right* | 0.568 | 0.593 | 0.486 | 0.498 |
|  | (0.226) | (0.238) | (0.196) | (0.200) |
| *Center* | 0.833 | 0.812 | 0.489 | 0.507 |
|  | (0.320) | (0.314) | (0.198) | (0.208) |
| *Center Left* | 0.883 | 0.844 | 0.870 | 0.863 |
|  | (0.310) | (0.300) | (0.354) | (0.355) |
| *Left* | 1.857 | 1.876 | 1.429 | 1.421 |
|  | (0.684) | (0.699) | (0.580) | (0.578) |
| *Independent* | 1.203 | 1.237 | 1.953 | 1.975 |
|  | (0.550) | (0.570) | (1.060) | (1.074) |
| *None* | 1.056 | 1.074 | 0.914 | 0.922 |
|  | (0.305) | (0.314) | (0.319) | (0.322) |
| Trust in police/military (reference=A lot) |  |  |  |  |
| *Quite some* | 1.200 | 1.206 | 1.557 | 1.543 |
|  | (0.367) | (0.375) | (0.410) | (0.413) |
| *Not a lot* | 4.889*** | 5.028*** | 3.113*** | 3.146*** |
|  | (1.525) | (1.597) | (0.792) | (0.811) |
| *No trust* | 16.29*** | 16.47*** | 8.362*** | 8.232*** |
|  | (5.356) | (5.517) | (2.291) | (2.284) |
| Authoritarianism | 1.447*** | 1.443*** | 1.417*** | 1.427*** |
|  | (0.116) | (0.117) | (0.121) | (0.124) |
| Security over liberties | 0.947* | 0.947* | 0.973 | 0.974 |
|  | (0.0251) | (0.0251) | (0.0266) | (0.0269) |
| Socioeconomic sector (reference=Highest (ABC1)) |  |  |  |  |
| C2 | 0.936 | 0.884 | 1.058 | 1.025 |
|  | (0.283) | (0.267) | (0.394) | (0.382) |
| C3 | 0.663 | 0.642 | 1.164 | 1.145 |
|  | (0.189) | (0.181) | (0.416) | (0.406) |
| D | 0.789 | 0.749 | 1.426 | 1.404 |
|  | (0.248) | (0.234) | (0.547) | (0.538) |
| Lowest (E) | 0.625 | 0.655 | 1.386 | 1.419 |
|  | (0.650) | (0.682) | (1.607) | (1.640) |
| Rural | 1.220 | 1.208 | 1.063 | 1.072 |
|  | (0.260) | (0.260) | (0.273) | (0.280) |
| Religion (reference=Protestant) |  |  |  |  |
| *Catholic* | 1.041 | 1.039 | 1.364 | 1.346 |
|  | (0.189) | (0.191) | (0.266) | (0.267) |
| *Other* | 0.848 | 0.813 | 2.496 | 2.375 |
|  | (0.573) | (0.572) | (1.354) | (1.273) |
| *None* | 1.321 | 1.344 | 1.487 | 1.478 |
|  | (0.269) | (0.276) | (0.322) | (0.324) |
| Education (reference=less than high school) |  |  |  |  |
| *Complete high school* | 1.179 | 1.181 | 1.087 | 1.076 |
|  | (0.222) | (0.227) | (0.200) | (0.202) |
| *Some college* | 1.049 | 1.060 | 1.052 | 1.052 |
|  | (0.260) | (0.265) | (0.271) | (0.275) |
| *Complete college* | 0.824 | 0.821 | 0.777 | 0.780 |
|  | (0.195) | (0.195) | (0.184) | (0.190) |
| *Graduate education* | 0.551 | 0.582 | 0.861 | 0.878 |
|  | (0.198) | (0.211) | (0.380) | (0.382) |
| Female | 1.682*** | 1.730*** | 1.604*** | 1.628*** |
|  | (0.237) | (0.245) | (0.214) | (0.220) |
| Age | 0.993 | 0.994 | 0.984*** | 0.984*** |
|  | (0.00433) | (0.00470) | (0.00415) | (0.00447) |
| Cut 1 | 0.385 | 0.373 | 0.796 | 0.843 |
|  | (0.267) | (0.263) | (0.580) | (0.619) |
| Cut 2 | 1.430 | 1.406 | 2.441 | 2.597 |
|  | (0.978) | (0.974) | (1.760) | (1.890) |
| Cut 3 | 13.59*** | 13.79*** | 15.58*** | 16.47*** |
|  | (9.458) | (9.729) | (11.31) | (12.08) |
| Cut 4 | 71.96*** | 72.74*** | 63.89*** | 67.49*** |
|  | (50.87) | (52.12) | (46.96) | (50.11) |
| Observations | 1,228 | 1,206 | 1,209 | 1,188 |
| Robust standard errors in parentheses. Odds ratios reported instead of coefficients. *** p<0.001, ** p<0.01, * p<0.05. | | | | |

**Table A2**

*Linear regressions of perceptions of frequency of human rights violations by the police and military during the October crisis.*

| Variables | Police | | Military | |
| --- | --- | --- | --- | --- |
| Demonstrated during the unrest | 0.206** | 0.202** | 0.159 | 0.149 |
|  | (0.0681) | (0.0678) | (0.0871) | (0.0877) |
| Media frequency use (all) | 0.122* |  | 0.0680 |  |
|  | (0.0591) |  | (0.0699) |  |
| Traditional media frequency use |  | 0.00883 |  | -0.0128 |
|  |  | (0.0618) |  | (0.0756) |
| Social media frequency use |  | 0.108* |  | 0.0819 |
|  |  | (0.0536) |  | (0.0674) |
| Worried about being victim of crime | 0.0290* | 0.0288* | 0.0343* | 0.0356* |
|  | (0.0113) | (0.0113) | (0.0146) | (0.0148) |
| Nearby violent protests | -0.00374*** | -0.00377*** | -0.00202 | -0.00206* |
|  | (0.000913) | (0.000916) | (0.00104) | (0.00104) |
| Ideology (reference=Right) |  |  |  |  |
| *Center Right* | -0.304 | -0.283 | -0.413 | -0.393 |
|  | (0.197) | (0.197) | (0.236) | (0.236) |
| *Center* | -0.0908 | -0.0982 | -0.424 | -0.398 |
|  | (0.180) | (0.181) | (0.225) | (0.228) |
| *Center Left* | -0.104 | -0.128 | -0.0998 | -0.0967 |
|  | (0.165) | (0.167) | (0.215) | (0.217) |
| *Left* | 0.169 | 0.169 | 0.172 | 0.173 |
|  | (0.157) | (0.157) | (0.214) | (0.215) |
| *Independent* | 0.0126 | 0.0141 | 0.238 | 0.244 |
|  | (0.192) | (0.193) | (0.275) | (0.275) |
| *None* | -0.0180 | -0.00974 | -0.103 | -0.0924 |
|  | (0.139) | (0.139) | (0.191) | (0.192) |
| Trust in police/military (reference=A lot) |  |  |  |  |
| *Quite some* | 0.135 | 0.142 | 0.251 | 0.246 |
|  | (0.155) | (0.156) | (0.148) | (0.150) |
| *Not a lot* | 0.869*** | 0.881*** | 0.631*** | 0.637*** |
|  | (0.153) | (0.155) | (0.140) | (0.141) |
| *No trust* | 1.341*** | 1.343*** | 1.166*** | 1.155*** |
|  | (0.155) | (0.156) | (0.147) | (0.148) |
| Authoritarianism | 0.164*** | 0.162*** | 0.201*** | 0.206*** |
|  | (0.0338) | (0.0340) | (0.0458) | (0.0465) |
| Security over liberties | -0.0161 | -0.0162 | -0.0124 | -0.0123 |
|  | (0.0115) | (0.0115) | (0.0146) | (0.0148) |
| Socioeconomic sector (reference=Highest (ABC1)) |  |  |  |  |
| C2 | -0.0328 | -0.0582 | 0.0879 | 0.0663 |
|  | (0.137) | (0.137) | (0.212) | (0.213) |
| C3 | -0.157 | -0.166 | 0.156 | 0.148 |
|  | (0.128) | (0.126) | (0.204) | (0.203) |
| D | -0.0587 | -0.0746 | 0.277 | 0.273 |
|  | (0.138) | (0.137) | (0.216) | (0.217) |
| Lowest (E) | -0.263 | -0.241 | 0.421 | 0.428 |
|  | (0.496) | (0.496) | (0.576) | (0.578) |
| Rural | 0.0477 | 0.0364 | -0.0379 | -0.0373 |
|  | (0.0909) | (0.0914) | (0.136) | (0.138) |
| Religion (reference=Protestant) |  |  |  |  |
| *Catholic* | 0.0331 | 0.0370 | 0.168 | 0.161 |
|  | (0.0832) | (0.0838) | (0.110) | (0.112) |
| *Other* | -0.0316 | -0.0542 | 0.438 | 0.404 |
|  | (0.267) | (0.271) | (0.295) | (0.293) |
| *None* | 0.135 | 0.145 | 0.183 | 0.181 |
|  | (0.0887) | (0.0891) | (0.121) | (0.122) |
| Education (reference=less than high school) |  |  |  |  |
| *Complete high school* | 0.0705 | 0.0687 | 0.0496 | 0.0413 |
|  | (0.0809) | (0.0821) | (0.104) | (0.106) |
| *Some college* | 0.0271 | 0.0316 | 0.0201 | 0.0184 |
|  | (0.103) | (0.104) | (0.142) | (0.145) |
| *Complete college* | -0.0551 | -0.0576 | -0.164 | -0.164 |
|  | (0.100) | (0.101) | (0.130) | (0.133) |
| *Graduate education* | -0.208 | -0.185 | -0.120 | -0.108 |
|  | (0.149) | (0.148) | (0.218) | (0.216) |
| Female | 0.218*** | 0.229*** | 0.239*** | 0.248*** |
|  | (0.0600) | (0.0601) | (0.0720) | (0.0727) |
| Age | -0.00410* | -0.00369 | -0.0100*** | -0.00943*** |
|  | (0.00193) | (0.00207) | (0.00236) | (0.00251) |
| Constant | 1.317*** | 1.312*** | 0.998** | 0.957* |
|  | (0.300) | (0.304) | (0.376) | (0.379) |
| Observations | 1,228 | 1,206 | 1,209 | 1,188 |
| R-squared | 0.378 | 0.384 | 0.301 | 0.302 |
| Robust standard errors in parentheses. *** p<0.001, ** p<0.01, * p<0.05. | | | | |
